# Study of Associated Genetic Variants in Indian Subjects Reveals the Basis of Ethnicity Related Differences in Susceptibility to Venous Thromboembolism

**DOI:** 10.1155/2014/182762

**Published:** 2014-09-30

**Authors:** Babita Kumari, Swati Srivastava, Tathagat Chatterjee, Rig Vardhan, Tarun Tyagi, Neha Gupta, Anita Sahu, Khem Chandra, Mohammad Zahid Ashraf

**Affiliations:** ^1^Defence Institute of Physiology & Allied Sciences, Timarpur, Delhi 110054, India; ^2^Army Hospital [R & R], Delhi Cantonment, New Delhi 110010, India; ^3^Genomics Division, Defence Institute of Physiology & Allied Sciences, Lucknow Road, Timarpur, Delhi 110054, India

## Abstract

The genetic variants linked with the susceptibility of individuals to VTE are well known; however, the studies explaining the ethnicity based difference in susceptibility to VTE are limited. Present study assesses mutations in six candidate genes contributing to the etiology of VTE in Indian subjects. The study comprised 93 VTE patients and 102 healthy controls. A PCR-RFLP based analysis was performed for nine mutations in the following genes associated with VTE: favtor V Leiden (FVL), prothrombin, tissue factor pathway inhibitor (TFPI), fibrinogen-beta, plasminogen activator inhibitor 1 (PAI-1), and methylene tetrahydrofolatereductase (MTHFR). All the subjects were found to be monomorphic for FVL 1691G/A, prothrombin 20210G/A and TFPI −536C/T mutations. The mutation in the MTHFR gene (677C/T) was observed only in patients. Contrarily, higher frequency of mutation in the PAI-1 −844G/A and the fibrinogen-*β* −455G/A was observed in controls in comparison to the patients. This study suggests that the PAI-1 −844G/A and fibrinogen-*β* −455G/A could be protective variants against VTE in Indians. While MTHFR 677C/T mutation was found to be associated, in contrast to other populations, the established genetic variants FVL 1691G/A, prothrombin 20210G/A, and TFPI −536C/T may not be associated with VTE in Indians thus revealing the basis of ethnicity related differences in susceptibility of Indians to VTE.

## 1. Introduction

Venous thrombosis may arise as a result of alterations in coagulation pathways, natural anticoagulants, or fibrinolytic mechanisms at cellular and molecular levels. The deficiencies of natural anticoagulants in plasma such as protein C [1], protein S, and antithrombin III as well as mutations in genes involved in coagulation have also been documented as common genetic risk factors for venous thrombosis [2]. Mutations in genes which include factor V Leiden (FVL), prothrombin (Factor II), methylene tetrahydrofolatereductase (MTHFR), plasminogen activator inhibitor-1 (PAI-1), fibrinogen-*β*, tissue factor pathway inhibitor (TFPI), and others have been described to understand the molecular basis of the risk of VTE. The genetic factors have been suggested to account for up to 60% of the risk of VTE, which comprises mainly the clinically evaluated SNPs including FVL 1691G/A and prothrombin 20210G/A.

These SNPs are prevalent in European ancestral population [3, 4] as compared to other populations worldwide. The significance of these genetic factors is highlighted by the fact that the incidence rate of VTE differs amongst different ethnicities globally [5]. Although with limited epidemiological data available on VTE in Indian population, a lower incidence of VTE had been reported compared with Caucasian, Mediterranean, or African counterparts [6, 7]. The annual incidence of idiopathic VTE in adults has been estimated to be approximately 23 per 100,000 among Caucasians, 29 per 100,000 among African Americans, and 14 per 100,000 among Hispanics compared to 6 per 100,000 among Asian-Pacific population [6]. The overall incidence of VTE has been estimated to be around 5 per 100,000 persons in Indian population with 64% being nonsurgical nontrauma patients [8]. While studying the high altitude induced DVT, we have recently found lack of factor VL and prothrombin 20210G/A mutations in Indian patients; however, the sample size was very small due to lower inhabitability and accessibility of the region [9].

The role of racial and ethnic factors has been proposed in determining the varying rate of VTE among different populations around the world [5, 10]. Although, there are considerable studies on prevalence of VTE and common associated genetic variants in European and American populations, the data from Asia are sparse. Therefore, in an attempt to understand the genetic basis for ethnicity related risk of developing VTE, a comprehensive study was designed to analyze the prevalence of nine clinically established SNPs in six genes in the Indian VTE patients.

In present study, we have observed that the three of the common SNPs of VTE, that is, FVL 1691G/A (rs6025), prothrombin 20210G/A (rs1799963), and TFPI −536C/T, were absent in our study population. However, the frequencies for mutations in MTHFR 677C/T (rs1801133) were observed only in patients and not in controls. On contrary, a higher frequency of the PAI-1 −844G/A (rs2227631) and the fibrinogen-*β* −455G/A (rs18000790) were observed in controls than in the patients. A comprehensive analysis for these mutations and their confounding effects had not been performed in Indian VTE patients before.

## 2. Materials and Methods

### 2.1. Subjects under Study

*Cases*. Ninety-three male patients from the Department of Pathology at Army Hospital R&R, Delhi (a tertiary care hospital), were approached to participate in this study. The written and informed consent of all patients was obtained before beginning the study procedures and the study was conducted in accordance with ethical standards of Helsinki declaration. All the patients were under 45 years of age and had either (DVT)/cerebral venous thrombosis, (CVT)/portal venous thrombosis, (PVT)/superior sagittal thrombosis (SST), and/or pulmonary thromboembolism (PE). The current protocol and consent procedure was approved by the human research ethical committees of Indian Council of Medical Research and Director General of Armed Forces Medical Sciences, India. Patients with the preexisting systemic diseases like malignancy, sickle cell anemia, vasculitis, and paroxysmal nocturnal hemoglobinuria were not included. All the patients had the diagnosis confirmed by at least one of the objective neuroimaging or radiological imaging methods. The clinical profile recording was done for each patient including the baseline demographic data (age, BMI, etc.). History regarding the presence of traditional risk factor like hypertension, diabetes mellitus, hyperlipoproteinemias, family history of bleeding disorders, smoking, past surgery or trauma, and so forth was also documented.

*Controls*. One hundred two healthy, age- and sex-matched subjects were selected as controls for this study. Baseline demographic data and history regarding mentioned factors were recorded for each subject. Subjects with any history of risk factors were not included in the study. The protocol and the consent procedures followed were same as those for patients.

### 2.2. Initial Laboratory Investigations

Initial hematological investigations including complete haemogram and coagulation screening comprising prothrombin time and activated partial thromboplastin time were carried out by standard methods. Other clinical parameters such as lipid profile, protein C, protein S, antithrombin III, and vasculitis profile were also recorded.

### 2.3. Restriction Fragment Length Polymorphism (RFLP) Based Genotyping

For genetic analysis peripheral blood samples were collected in EDTA vacutainers. High molecular weight DNA was extracted from the peripheral blood by QIAamp DNA isolation kit (Qiagen, Germany), using manufacturer's protocol. Quantitative analysis of genomic DNA was done using DNA/RNA nanodrop 2000 spectrophotometer (Thermo Fischer, USA). For qualitative analysis, the samples (100 ng) were loaded on 0.7% agarose gel containing ethidium bromide and run for ~20 min and visualized under UV. The desired gene sequences of FVL, prothrombin, MTHFR, PAI-1, fibrinogen-*β*, and TFPI genes were amplified using specific PCR primers (as detailed in Table 1). The final PCR reaction contained 100 ng of DNA, 200 *μ*M deoxyribonucleotide triphosphate (dNTP), 10 pmol of each primer, and 0.6 U* taq* polymerase in total volume of 25 *μ*L reaction buffer (50 mM KCL, 20 mM Tris-HCL, pH 8.3). The amplified PCR products were digested with specific restriction enzymes at optimized temperature. The digested PCR products were mixed with DNA loading dye and subjected to agarose gel electrophoresis at varying concentrations ranging from 1.5 to 3.5% depending upon the band sizes to be obtained after digestion. For each experiment, SNP-positive (sample with known profile) and SNP-negative (blank) samples were used for verifying results. Complete details of PCR conditions and the genotypes screened for different genes are listed in Table 1.

### 2.4. Statistical Analysis

The genotypic and allelic frequencies were determined by gene counting and compared by the 3 × 2 and 2 × 2 contingency table, respectively. The genotypic distributions were compared between the study groups by chi-square test (degree of freedom = 2) and Fisher's exact test and the risk assessment was done by calculation of odd's ratio (OR) (with 95% confidence interval (CI)) using Prism 5.0 (Graphpad, USA). The *P* value lesser than 0.05 was the criteria for significance for all statistical tests. Percentage of the heterozygosity, *t*-test between expected and observed heterozygosity, and Hardy-Weinberg equilibrium (one degree of freedom) from the observed distribution of genotypes in the population as well as linkage disequilibrium were calculated using the R core team (2012): R Foundation of Statistical Computing, Vienna, Austria (ISBN 3-900051-07-0, http://www.R-project.org).

## 3. Results

### 3.1. Clinical Characteristics of Patients

The mean age of patients was 32 years (SD ± 3.96). No significant difference in the mean age of patients and controls was observed. All the subjects (patients and controls) were physically active and the BMI for each of them was in normal range (<29.9 Kg/m^2^). Out of 93 patients, 52 were diagnosed to have DVT while others had CVT, PVT, SST, or PE. Most of the patients had normal haemogram and 21 patients were found to be hyperlipidemic. Additionally, we analyzed the contribution of other provoking factors such as low protein C, protein S, and antithrombin III. Of all the patients, 32% had protein C deficiency (<70% of activity), 47% had low protein S levels (<65% activity), and 25% had decreased antithrombin III levels (<80% activity).

### 3.2. Genotypic Frequencies of FVL, Prothrombin, and TFPI Genes

The study group was found to be monomorphic for mutations in FVL 1691G/A (rs6025), prothrombin 20210G/A (rs1799963), and TFPI (−536C/T) genes (Table 2). All three polymorphic variants were absent from both the patients and the control groups, as no restriction site was obtained in the corresponding amplified PCR products. The patient and control groups were similarly genotyped as 1691GG, 20210GG, and −536CC for the three genes FVL, prothrombin, and TFPI, respectively.

### 3.3. Genotypic Frequencies of PAI-1, MTHFR, and Fibrinogen-*β* Gene

Other three genes (PAI-1, MTHFR, and Fibrinogen-*β*) previously reported to be involved in pathogenesis of VTE were analysed for the six known SNPs, that is, −844G/A (rs2227631) and −675 4G/5G (rs1799889) for PAI-1, 677C/T (rs1801133) and 1298A/C (rs1801131) for MTHFR, and −455G/A (rs18000790) and 148C/T (rs1800787) for fibrinogen-*β* chain. Two of the SNPs, that is, −675 4G/5G and −844G/A in PAI-1, gene were analysed in the present study. The RFLP analysis demonstrated no significant difference between the control and the patient groups for −675 4G/5G polymorphism both at the genotypic (*χ* ^2^ = 2.01 and *P* = 0.36) as well as at the allelic level (*P* = 0.76, OR = 0.92, and CI = 0.62–1.38; Table 3), whereas the prevalence of −844G/A polymorphism of PAI-1 was significantly different at the genotypic level (*χ* ^2^ = 10.99 and *P* = 0.004). The frequency of this polymorphism was observed to be significantly lesser in patients (1.07%) compared to the control subjects (13.72%) while no significant difference was observed at the allelic level.

Next, we analysed two common SNPs, that is, 677C/T and 1298A/C in MTHFR gene. The distribution of 677C/T genotype was observed to be statistically different between the two groups (*P* = 0.02, OR = 0.63, and CI = 0.35–1.12; Table 3). A higher percentage of CC was observed in the patient group (78.49%) than that of control group (62.04%). The component of heterozygosity was higher in controls (34.95%) compared to the patients group (19.35%); however, the difference for the allelic frequencies was not significant. Further, the frequency of homozygous recessive genotype (mutant) 677TT was very low and was observed in only two patients. There was no significant difference in prevalence of other SNP for the MTHFR gene, 1298A/C, between the patients and controls (Table 2).

Amongst the two SNPs −455G/A and 148C/T studied for fibrinogen-*β* chain gene, the −455G/A was found only in control subjects while it was completely absent from the patients (*χ* ^2^ = 6.93 and *P* = 0.003, Table 3). The frequency was 6.68% in controls compared to 0% in patients. The frequencies of the wild type genotype, that is, −455GG, and the heterozygous genotype, that is, −455GA, amongst the two groups were comparable whereas the frequency distribution of the alleles was not significantly different. The other SNP of fibrinogen-*β* chain, that is, 148C/T, showed no statistically significant difference at the genotypic or the allelic levels.

### 3.4. Heterozygosity Test and Linkage Disequilibrium Analysis

**Figure 1:**
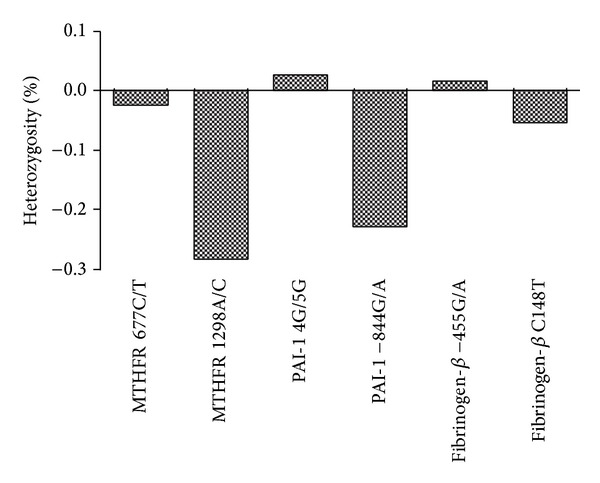
The percent heterozygosity of SNPs under study. As mentioned in Section 2, paired *t*-test was carried out between expected and observed heterozygosities of the SNPs under study. The overall difference in expected and observed heterozygosity values was not found to be significant (*P* = 0.07).

The observed heterozygosity was much higher than expected for the SNPs: PAI-1 −844G/A and MTHFR 1298A/C (Figure 1). Paired *t*-test was carried out between the expected and the observed heterozygosities. The overall difference in expected and observed heterozygosity values was not found to be significant (*t* = −1.6, df = 8, and *P* = 0.07).

**Figure 2:**
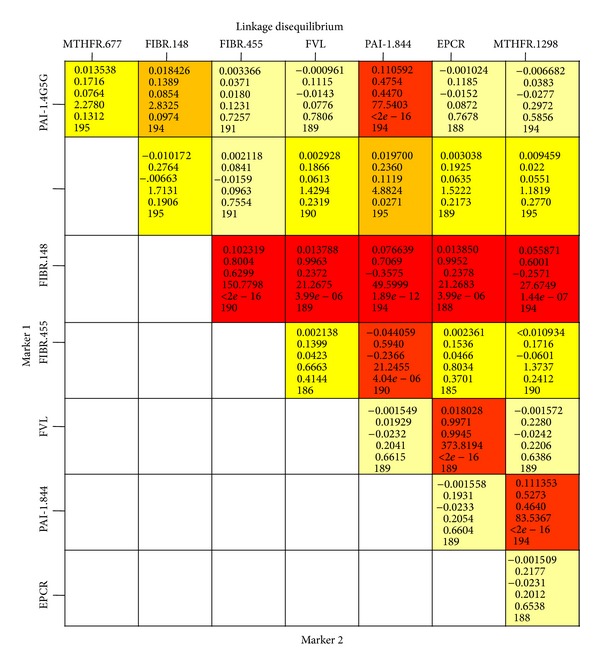
Linkage disequilibrium (LD) analysis among SNPs. The red and orange boxes are the loci pairs which are significantly linked with each other. The relevant variables for multivariate analysis were selected on the basis of their association found in univariate analysis and linkage of variables.

The linkage disequilibrium data revealed a significant association between the variables PAI-1 −844G/A and with each of the five SNPs including PAI-1 4G/5G, MTHFR 1298A/C, MTHFR 677C/T, fibrinogen-*β*  −455G/A, and fibrinogen-*β* 148C/T. The linkage disequilibrium was also observed for the fibrinogen-*β* 148C/T with fibrinogen-*β*  −455G/A and the MTHFR 1298A/C (Figure 2). The relevant variables for multivariate analysis were selected on the basis of their association found in univariate analysis and the linkage of variables.

## 4. Discussion

Associations between certain genetic variants, mainly in the proteins of the coagulation system, fibrinolytic factors, and platelet membrane receptors, and the epidemiology for VTE have been suggested in different populations globally [11, 12]. In the present study we investigated nine most common SNPs in factor V, prothrombin, TFPI, MTHFR, PAI-1, and fibrinogen-*β* chain genes, to understand the link between the common genetic variations in the genes involved and the incidence of VTE disorders in Indian subjects. The common clinical parameters, such as lipid profile, protein C, protein S, antithrombin III, did not show any association with the presence of any particular polymorphism.

Mutations in genes factor V (Leiden) and prothrombin (20210G/A) are the most common genetic variations leading to hypercoagulable state and are thought to be responsible for the increased susceptibility to VTE in general. Even the heterozygous and the homozygous carriers of the FVL1691 G/A mutation have an increased risk of venous thrombosis compared with noncarriers [12]. Accordingly, a higher rate of FV1691G/A and/or the prothrombin 20210G/A polymorphisms has been observed in VTE patients among different populations around the world [13, 14]. In the present study none of the subjects (patients or controls) was found to be positive for either FVL 1691G/A or prothrombin 20210G/A. Previously, few Indian studies have observed very low frequency of FV1691G/A polymorphism while a complete absence of prothrombin 20210G/A [15–17]. These include a study on the healthy Indians in Malaysia which showed only 5.6% heterozygous individuals while no homozygous individual for the FVL mutation (1691G/A) and none of the subjects to have prothrombin gene mutation 20210G/A [16]. Thus theresults of the present study and previous reports suggest that both of these polymorphisms, which are commonly observed in VTE patients in other parts of the world, have a limited role in imparting susceptibility to thromboembolic disorders at least in Indians.

The lower levels of TFPI protein have been associated with increased risk of venous thrombosis [18]. In an earlier study, the genetic mutation −536C/T (Pro151Leu) was claimed to be associated with an increased risk of VTE without affecting the plasma activity or concentration of TFPI [19]. In contrast to this, in present study, we found that the −536C/T mutation in the TFPI gene was not present in any of the subjects. To the best of our knowledge, this is the first study on TFPI mutation in Indians and needs to be performed with a larger sample size in future.

Another important factor of coagulation, PAI-1, acts as a major inhibitor of tissue type plasminogen activator (tPA) and thus increases in PAI-1 levels results in poor fibrinolytic capacity and had been linked with the pathogenesis of thrombosis [20]. A deletion/insertion (4G/5G) polymorphism at 675 upstream from the transcription initiation site in the promoter lesion of PAI-1 gene is reportedly associated with the plasma PAI-1 levels. The 4G allele has been shown to be coupled with the higher transcription rate and consequently elevated PAI-1 plasma levels thereby possibly increasing the risk for thrombosis [21]. However, we did not find any significant difference between the patients and the control subjects for PAI-1 −675 4G/5G, which may be due to the difference of ethnicity in our study population. We also investigated another important SNP (−844G/A) of PAI-1. A significantly higher percentage of −844G/A mutants in control group (13.72%) compared to patients (1.07%) (*P* = 0.004 and *χ* ^2^ =10.99) suggested a protective role of this mutation in our study group.

Mutations in the MTHFR gene have been associated with the hyperhomocysteinemia which is in turn considered as a risk factor for thrombosis, coronary artery disease, and atherosclerosis [22, 23]. The 677C/T is a well-described mutation of this gene affects the homocysteine metabolism that results in hyperhomocysteinemia which leads to the substitution of alanine to valine at position 226 in the protein [24]. We also observed a significant difference in the frequency of 677C/T polymorphism in MTHFR gene between the patients and the controls, although the frequency for homozygous recessive form (mutants) was very low, that is, only two cases in patients' group. Though, the 677TT genotype, associated with the elevated homocysteine levels, was absent in controls, a higher incidence of heterozygosity (34.9%) was observed in these subjects. The frequency of MTHFR 677C/T polymorphism varies to a great extent in different ethnic populations [25]. Mild hyperhomocysteinemia has been univocally accepted as an independent predictor of the cardiovascular diseases [26, 27]. However, the role of homocysteine in directly modulating the risk of cardiovascular diseases has been recently challenged, which has transformed the homocysteine hypothesis into the homocysteine controversy [28]. This controversy has arisen because of the contradicting results obtained by later studies which found no consistent effect of the T allele on risk of cardiovascular disease occurrence [29, 30]. Collective evidence shows that the administration of high dose multi-B vitamins to patients with (a high risk of) CVD could not produce the beneficial effects, whereas another SNP (1298A/C) evaluated in MTHFR gene which leads to the substitution of glutamate to alanine in MTHFR protein [31] was comparable in both the groups.

Elevated fibrinogen levels in plasma have been implicated for the increased risk of vascular events including myocardial infarction, stroke, and VTE [32, 33]. The synthesis of the fibrinogen-*β* chain is the rate limiting step in the synthesis of fibrinogen [33]. Studies have confirmed that the mutations in fibrinogen-*β* gene, the 148C/T polymorphism remains in complete linkage disequilibrium with the −455G/A polymorphism [34, 35]. Our findings are at variance with these reports; 148C/T polymorphism of the fibrinogen-*β* gene did not show a significant difference between the patients and controls. However, for −455G/A SNP, significant difference was observed between the two groups. The mutant AA genotype was only observed in controls (6.86%) but not in patients (0%). These discrepancies could be attributed to the ethnic factor and further question the role of the known genetic variants, prevalent in western countries, in pathogenesis of VTE in Indian population.

## 5. Conclusion

The absence of SNPs in three prominent genes (FVL, prothrombin, and TFPI) suggests the limited role of established genetic variants in imparting susceptibility to VTE in Indian population. Interestingly, while the MTHFR 677C/T mutation appears to contribute towards VTE susceptibility, the PAI-1 −844G/A and the fibrinogen-*β* −455G/A variants might be playing a protective role. These results, thus, encourage the identification of novel genetic variants in Indians and other Asian populations for better understanding of the ethnicity based differences in susceptibility of the individuals to VTE.

## Figures and Tables

**Table 1:** PCR and genotyping details.

Gene	Polymorphism, region	Primer details	PCR product (bp)	Annealing temperature	Restriction enzyme	Band size (bp)
Factor V Leiden	1691 G/A, rs6025	F: TCAGGCAGGAACAACACCAT R: GGTTACTTCAAGGACAAAATACCTGTAAAGCT	241	58°C	*Hind III *	G = 241 A = 209, 32

Prothrombin	20210G/A, 3′UTR rs1799963	F: ATTGATCAGTTTGGAGAGTAGGGG R: AATAGCACTGGGAGCATTGAAGCT	142	60°C	*Hind III *	G = 142 A = 119, 23

TFPI	−536C/T, intron7	F: TCTATTTTAATTGGCTGTAT R: GCATGATAATAGTTTCCTGG	170	65°C	*BseN1 *	C = 170 T = 143, 27

PAI-1	4G/5G, −675 promoter rs1799889	F: CACAGAGAGAGTCTGGCCACGT R: CCAACAGAGGACTCTTGGTCT	98	60°C	*Bsl I *	4G = 98 5G = 77, 22

PAI-1	−844G/A, 3′UTR rs2227631	F: CAGGCTCCCACTGATTCTAC R: GAGGGCTCTCTTGTGTCAAC	510	60°C	*XhoI *	G = 510 A = 364, 146

MTHFR	677C/T, rs1801133	F: TGAAGGAGAAGGTGTCTGCGGGA R: AGGACGGTGCGGTGAGA	198	62°C	*Hinf I *	C = 198 T = 175, 23

MTHFR	1298A/C, rs1801131	F: CTTTGGGGAGCTGAAGGACTACTAC F: CAATTTGTGACCATTCCGGTTTG	163	62°C	*MboII *	A = 56, 31, 30, 28, 18 C = 84, 31, 30, 18

Fibrinogen-*β*	148C/T, promoter rs1800787	F: CCTAACTTCCCATCATTTTGTCCAATAAA R: TGTCGTTGACACCTTGGGACTTAACTAG	362	53°C	*Hind III *	C = 265, 97 T = 362

Fibrinogen-*β*	−455G/A, promoter rs18000790	F: GCTTGTGGGAAATGAAGGAA R: GGCAACCACTAAAATCGTGA	469	59.5°C	*HaeIII *	A = 469, 26 G = 383, 86, 26

PCR1-RFLP details showing primer sequences, PCR product size, annealing temperature, restriction enzyme, digestion temperature, and the fragment sizes.

**Table 2:** Genotypic and allelic distribution.

Serial number	Gene	Study group	Genotype (frequency)			Allele (frequency)		HWE (*P* value)
			GG	GA	AA	G	A	

1	FVL 1691G/A	Control	102 (100)	0	0	204 (100)	0	1.0
		Patients	93 (100)	0	0	186 (100)	0	1.0

			GG	GA	AA	G	A	

2	Prothrombin 20210G/A	Control	102 (100)	0	0	204 (100)	0	1.0
		Patients	93 (100)	0	0	186 (100)	0	1.0

			CC	CT	TT	C	T	

3	TFPI −536C/T	Control	102 (100)	0	0	204 (100)	0	1.0
		Patients	93 (100)	0	0	86 (100)	0	1.0

			4G	4G/5G	5G	4G	5G	

4	PAI-1 −6754G/5G	Control	27 (26.47)	53 (51.96)	22 (21.56)	107 (52.45)	97 (47.75)	0.67
		Patients	31 (33.33)	39 (41.93)	23 (24.73)	101 (54.30)	85 (45.69)	0.13

			GG	GA	AA	G	A	

5	PAI-1 −844G/A	Control	20 (19.6)	68 (66.66)	14 (13.72)	108 (52.94)	112 (60.21)	**0.0006**
		Patients	20 (21.50)	72 (77.41)	1 (1.07)	96 (47.05)	74 (39.78)	0

			CC	CT	TT	C	T	

6	MTHFR 677C/T	Control	66 (62.04)	36 (34.95)	0	170 (82.52)	36 (17.47)	**0.03**
		Patients	73 (78.49)	18 (19.35)	2 (2.15)	164 (88.17)	22 (11.82)	0.48

			AA	AC	CC	A	C	

7	MTHFR 1298A/C	Control	18 (16.66)	84 (82.35)	0	120 (58.82)	84 (41.17)	0
		Patients	31 (33.33)	62 (66.66)	0	124 (66.66)	62 (33.33)	0

			CC	CT	TT	C	T	

8	Fibrinogen-*β* 148C/T	Control	52 (50.98)	45 (44.11)	5 (4.90)	149 (73.03)	55 (26.96)	0.22
		Patients	53 (56.98)	38 (40.86)	2 (2.15)	144 (77.41)	42 (22.58)	0.10

			GG	GA	AA	G	A	

9	Fibrinogen-*β* −455 G/A	Control	71 (69.60)	24 (23.52)	7 (6.86)	166 (81.37))	38 (18.62)	**0.02**
		Patients	62 (69.66)	27 (30.33)	0	151 (84.83)	27 (15.16)	0.09

Observed frequency distribution of genotypic and allelic frequencies in the control (*N* = 102) and patients (*N* = 93) group along with HWE calculation. The *P* value lesser than 0.05 was the criteria for significance for all statistical tests.

**Table 3:** Statistical analysis for polymorphic SNPs.

Gene	*χ* ^2^ (df = 2)	*P* value	Fischer's exact	OR	CI
PAI-1 −6754G/5G	2.01	0.36	0.76	0.92	0.62–1.38
PAI-1 −844 G/A	10.99	**0.004**	0.15	0.74	0.49–1.11
MTHFR 677C/T	7.7	**0.02**	0.12	0.63	0.35–1.12
MTHFR 1298A/C	0	0	0.11	0.71	0.47–1.08
Fibrinogen-*β* −455G/A	6.93	**0.03**	0.41	0.78	0.45–1.34
Fibrinogen-*β* 148C/T	1.47	0.47	0.34	0.79	0.49–1.25

Statistical tests of SNPs in candidate genes. *χ* ^2^ test and observed *P* values show significant difference in MTHFR C677T, PAI −844 G/A, and fibrinogen-*β* chain −455G/A SNPs (*P* < 0.05). No SNP showed significant allelic difference between patients and control group (Fisher's exact >0.05). The *P* value lesser than 0.05 was the criteria for significance for all statistical tests.

## References

[1] Koster T, Rosendaal FR, Briet E (1995). Protein C deficiency in a controlled series of unselected outpatients: an infrequent but clear risk factor for venous thrombosis (Leiden Thrombophilia Study). *Blood*.

[2] Ridker PM, Miletich JP, Hennekens CH, Buring JE (1997). Ethnic distribution of factor V Leiden in 4047 men and women: implications for venous thromboembolism screening. *Journal of the American Medical Association*.

[3] Souto JC, Almasy L, Borrell M (2000). Genetic susceptibility to thrombosis and its relationship to physiological risk factors: the GAIT study. *The American Journal of Human Genetics*.

[4] Coon WW (1977). Epidemiology of venous thromboembolism. *Annals of Surgery*.

[5] White RH, Keenan CR (2009). Effects of race and ethnicity on the incidence of venous thromboembolism. *Thrombosis Research*.

[6] White RH, Zhou H, Romano PS (1998). Incidence of idiopathic deep venous thrombosis and secondary thromboembolism among ethnic groups in California. *Annals of Internal Medicine*.

[7] Kapoor VK (2010). Venous thromboembolism in India. *National Medical Journal of India*.

[8] Lee AD, Stephen E, Agarwal S, Premkumar P (2009). Venous Thrombo-embolism in India. *European Journal of Vascular and Endovascular Surgery*.

[9] Tyagi T, Ahmad S, Gupta N (2014). Altered expression of platelet proteins and calpain activity mediate hypoxia-induced prothrombotic phenotype.. *Blood*.

[10] Zakai NA, Mcclure LA (2011). Racial differences in venous thromboembolism. *Journal of Thrombosis and Haemostasis*.

[11] Seligsohn U, Lubetsky A (2001). Genetic susceptibility to venous thrombosis. *The New England Journal of Medicine*.

[12] Simioni P, Sanson B-J, Prandoni P (1999). Incidence of venous thromboembolism in families with inherited thrombophilia. *Thrombosis and Haemostasis*.

[13] Rosendorff A, Dorfman DM (2007). Activated protein C resistance and factor V Leiden: a review. *Archives of Pathology and Laboratory Medicine*.

[14] Margaglione M, Brancaccio V, De Lucia D (2000). Inherited thrombophilic risk factors and venous thromboembolism: distinct role in peripheral deep venous thrombosis and pulmonary embolism. *Chest*.

[15] Ghosh K, Shetty S, Madkaikar M (2001). Venous thromboembolism in young patients from Western India: a study. *Clinical and Applied Thrombosis/Hemostasis*.

[16] Abdullah WZ, Kumaraguru S, Ghazali S, Yusoff NM (2010). Factor V Leiden and prothrombin G20210A mutations among healthy Indians in Malaysia. *Laboratory Medicine*.

[17] Kumar SI, Kumar A, Srivastava S, Saraswat VA, Aggarwal R (2005). Low frequency of factor V Leiden and prothrombin G20210A mutations in patients with hepatic venous outflow tract obstruction in northern India: a case-control study. *Indian Journal of Gastroenterology*.

[18] Nekoo AA, Simon Futers T, Moia M, Mannucci PM, Grant PJ, Ariëns RAS (2001). Analysis of the tissue factor pathway inhibitor gene and antigen levels in relation to venous thrombosis. *The British Journal of Haematology*.

[19] Kleesiek K, Schmidt M, Götting C (1999). The 536C→T transition in the human tissue factor pathway inhibitor (TFPI) gene is statistically associated with a higher risk for venous thrombosis. *Thrombosis and Haemostasis*.

[20] Gonzalez-Conejero R, Lozano ML, Corral J, Martinez C, Vicente V (2000). The TFPI 536C→T mutation is not associated with increased risk for venous or arterial thrombosis. *Thrombosis and Haemostasis*.

[21] Dawson SJ, Wiman B, Hamsten A, Green F, Humphries S, Henney AM (1993). The two allele sequences of a common polymorphism in the promoter of the plasminogen activator inhibitor-1 (PAI-1) gene respond differently to interleukin-1 in HepG2 cells. *The Journal of Biological Chemistry*.

[22] Cattaneo M (1999). Hyperhomocysteinemia, atherosclerosis and thrombosis. *Thrombosis and Haemostasis*.

[23] Khare A, Ghosh K, Shetty S, Kulkarni B, Mohanty D (2004). Combination of thrombophilia markers in acute myocardial infarction of the young. *Indian Journal of Medical Sciences*.

[24] Frosst P, Blom HJ, Milos R (1995). A candidate genetic risk factor for vascular disease: a common mutation in methylenetetrahydrofolate reductase. *Nature Genetics*.

[25] Fletcher O, Kessling AM (1998). MTHFR association with arteriosclerotic vascular disease?. *Human Genetics*.

[26] Homocysteine Studies Collaboration (2002). Homocysteine and risk of ischemic heart disease and stroke: a meta-analysis. *The Journal of the American Medical Association*.

[27] Hansson GK (2005). Mechanisms of disease: inflammation, atherosclerosis, and coronary artery disease. *The New England Journal of Medicine*.

[28] Smulders YM, Blom HJ (2011). The homocysteine controversy. *Journal of Inherited Metabolic Disease*.

[29] Clarke R Evidence against a causal association of homocysteine and coronary heart disease: a mendelian randomization study.

[30] Lewis SJ, Ebrahim S, Smith GD (2005). Meta-analysis of MTHFR 677C→T polymorphism and coronary heart disease: does totality of evidence support causal role for homocysteine and preventive potential of folate?. *British Medical Journal*.

[31] Morange PE, Henry M, Tregouët D (2000). The A -844G polymorphism in the PAI-1 gene is associated with a higher risk of venous thrombosis in factor V leiden carriers. *Arteriosclerosis, Thrombosis, and Vascular Biology*.

[32] Wilhelmsen L, Svardsudd K, Korsan-Bengtsen K (1984). Fibrinogen as a risk factor for stroke and myocardial infarction. *The New England Journal of Medicine*.

[33] Koster T, Rosendaal FR, Reitsma PH, van der Velden PA, Briet E, Vandenbroucke JP (1994). Factor VII and fibrinogen levels as risk factors for venous thrombosis. *Thrombosis and Haemostasis*.

[34] Van'T Hooft FM, Von Bahr SJF, Silveira A, Iliadou A, Eriksson P, Hamsten A (1999). Two common, functional polymorphisms in the promoter region of the *β*- fibrinogen gene contribute to regulation of plasma fibrinogen concentration. *Arteriosclerosis, Thrombosis, and Vascular Biology*.

[35] Behague I, Poirier O, Nicaud V (1996). *β* Fibrinogen gene polymorphisms are associated with plasma fibrinogen and coronary artery disease in patients with myocardial infarction: the ECTIM study. *Circulation*.

